# Are anti-PD-1-associated immune related adverse events a harbinger of favorable clinical prognosis in patients with gastric cancer?

**DOI:** 10.1186/s12885-022-10199-x

**Published:** 2022-11-05

**Authors:** Xiaoyun Zhang, Shuo Xu, Jiaqi Wang, Yalei Lv, Na Wang, Ruixue Lai, Ziyue Sha, Qun Zhao, Zhanjun Guo

**Affiliations:** 1 Department of Rheumatology and Immuology, The Fourth Hospital of Hebei Medical University, 12 Jiankang Road, Shijiazhuang, 050011 People’s Republic of China; 2 School of Basic Medicine, Hebei Medical University, Shijiazhuang, People’s Republic of China; 3 Department of Medical Oncology, The Fourth Hospital of Hebei Medical University, Shijiazhuang, People’s Republic of China; 4 Department of Gastroenterology and Hepatology, The Fourth Hospital of Hebei Medical University, Shijiazhuang, People’s Republic of China; 5 Department of Surgery, The Fourth Hospital of Hebei Medical University, Shijiazhuang, People’s Republic of China

**Keywords:** Gastric cancer, Immune-related adverse events, Immune checkpoint inhibitors, Anti-PD-1 antibody, OS, PFS

## Abstract

**Background:**

Immune checkpoint inhibitors (ICIs) has shown remarkable benefit in the treatment of a range of cancer types, although it may initiate immune related adverse events (irAEs) in patients. Some studies have shown that there is a close relationship between the occurrence of irAEs and prognosis. In present study, we have attempted to establish whether the occurrence of irAEs after the use of anti PD-1 antibodies is associated with treatment efficacy in people with advanced gastric cancer (AGC).

**Methods:**

This study included patients treated with the anti-PD-1 antibodies for AGC patients at The Fourth Hospital of Hebei Medical University. IrAEs were identified clinically and graded as per the National Cancer Institute Common Terminology Criteria for Adverse Events ver. 4.03. Efficacy was evaluated with objective response rate (ORR), disease control rate (DCR), progression free survival (PFS) and overall survival (OS). The analysis was performed to determine the association between irAEs and clinical outcomes.

**Result:**

Of the 74 AGC patients in our study, 24 developed irAEs. The DCR of the irAE displayed a trend better than that of non-irAE group but without statistical difference (41.70% VS 6.0%, *p* = 0.118). Median PFS in the irAE group was superior to that in the non-irAE group (176 days VS 94 days, *p* = 0.001). Median OS also showed this trend of difference at borderline statistical level (292 days VS 239 days, *p* = 0.057). Multivariate analysis also demonstrated irAE (HR = 0.269, 95%CI: 0.088 to 0.822, *p* = 0.021) were associated independently with the better prognosis for AGC patients.

**Conclusion:**

In advanced gastric cancer treated with anti PD-1 antibodies, the occourence of irAEs might contribute to the improved prognosis.

**Supplementary Information:**

The online version contains supplementary material available at 10.1186/s12885-022-10199-x.

## Introduction

Although the morbidity and mortality rate of gastric cancer have been decreasing for the past decade, it remains an important cancer worldwide responsible for over 1,000,000 new cases with an estimated 769,000 deaths, according to new data published by GLOBOCAN 2020 [1]. Systemic chemotherapy is an important method to relieve symptoms and prolong survival time for unresectable or recurrent advanced gastric cancer (AGC). For patients with human epidermal growth factor receptor 2 (HER2)-negative AGC, multidrug combination chemotherapy combinations including fluoropyrimidine, platinum agent and taxane is standard first-line chemotherapeutic regimen. But in HER2-positive AGC, the combination of fluoropyrimidine, platinum agent, and trastuzumab (an anti-HER2 antibody) is standard first-line chemotherapeutic regimen [2]. Even so, the prognosis for AGC patients remains poor with median survival times of 10–13 months [3, 4].

Immune checkpoint inhibitors (ICIs), a type of drug that increase the cytotoxicity of T cells by blocking intrinsic downregulators of immunity such as cytotoxic T-lymphocyte antigen 4 (CTLA-4) and programmed cell death 1 (PD-1) or programmed cell death ligand 1 (PD-L1), have become a new dawn in cancer treatment recently [5]. Nivolumab over placebo has showed longer overall survival (OS) and lower risk of death in the ATTRACTION-2 study or in the further exploration (ATTRACTION-4 and CheckMate 649), nivolumab-chemotherapy combination was well tolerated and demonstrated encouraging efficacy [6–8]. But compared with chemotherapy in AGC patients with PD-L1 CPS ≥ 1 or ≥ 10, the combination of pembrolizumab and chemotherapy was not superior to chemotherapy for the OS and PFS end points tested [9]. What’s more, camrelizumab plus apatinib and pembrolizumab plus lenvatinib have showed promising anti-tumour activity with an acceptable safety profile in AGC patients [10, 11]. All these mentioned above indicate that ICIs have more and more widely used in gastric cancer.

Immune checkpoint inhibitors cause immune tolerance imbalance leading to inflammatory side effects known as immune-related adverse events (irAEs) [12]. The occurrence of immune-related adverse events was thought to be related to the disruption of immune homeostasis by immune checkpoint inhibitors. The mechanism of irAEs is unclear, but it considered be related to the following factors: increasing T-cell activity against antigens present in tumors and healthy tissue, increasing levels of preexisting autoantibodies or inflammatory cytokines and enhanced complement-mediated inflammation due to direct binding of an antibody against CTLA-4 [5]. IrAE is different from the system-specific or organ-specific adverse events associated with chemotherapy, it can occour in any organ of the body such as skin, gastrointestinal tract, lung and so on [13].

Some recent studies have shown that the occourence of irAEs maybe associated with better prognosis in patients with various cancers [14]. A previous study has shown an association between irAEs and prognosis in AGC patients treated with nivolumab [15]. However, this evidence is not sufficient in AGC patients treated with ICIs plus targeted medicine or chemotherapeutic medicine. Thus, we collected information from AGC patients treated with different anti-PD-1 antibody (monotherapy or combine with targeted medicine/chemotherapeutic medicine) and investigated the correlation between irAEs and ICIs efficacy in AGC patients.

## Methods

### Patients

AGC patients who received anti-PD-1 antibody (monotherapy or combine with targeted medicine/chemotherapeutic medicine) at The Fourth Hospital of Hebei Medical University from February 2019 to April 2020 were included in this study. Patients with a history of other immunotherapy were excluded. All data were collected retrospectively from electronic medical records. The clinical information we collected were as follows: age, gender, Eastern Cooperative Oncology Group performance status (ECOG PS), treatment line number, number of metastatic sites, histologic type, HER2 status, disease status, baseline blood cell count, alkaline phosphatase (ALP) and neutrophilto-lymphocyte ratio (NLR) before initiating treatment. IrAEs were defined as those inflammatory side effects which were caused by imbalances in immunological tolerance because of the immune checkpoint inhibitors. For the irAEs assessment, the National Cancer Institute Common Terminology Criteria for Adverse Events ver. 4.03 (https://ctep.cancer.gov/protocolDevelopment/electronic_applications/ctc.htm#ctc_40) was used and we assessed the irAEs by at least 2 or more clinical oncologists, one of whom was a senior-level physician. We divided the patients into two groups as irAE group and non-irAE group based on occurrence of irAEs and compared the efficacy between these two groups.

Due to the retrospective nature of this study and this analysis was conducted using only existing information, waiver of informed consent was applied for participating patients. All procedures were confirmed by the ethics committee of the Fourth Hospital of Hebei Medical University.

### Treatment and assessment

Patients received standard anti-PD-1 antibody (monotherapy or combine with targeted medicine/chemotherapeutic medicine) every 21 days until disease progression, clinical deterioration, unacceptable toxicity, or patient’s refusal. Type of anti-PD-1 antibodys, targeted medicine and chemotherapeutic medicine were listed in Table S1. After starting treatment, clinical and laboratory tests were carried out as clinically indicated each cycle before drug administration. Body computed tomography (CT) scans were taken every 2–3 cycles. Objective tumor response was evaluated according to the RECIST version 1.1.

### Statistical analysis

Classified variables were compared using the Fisher’s exact tests and Mann–Whitney U tests for continuous variables. For continuous variables, means and standard deviations (SDs) are shown in the case of variables have a normal distribution, and median and interquartile range (IQR) in the case of variables that do not. The PFS data were calculated from the first dose until progression or death or censured at the last date of the follow-up. The OS data were calculated from the diagnosis until death or censored at the last date of the follow-up. To determine the association between the incidence of irAEs and prognosis, ORR, DCR and Chisquared test were performed. The association between the presence of irAEs with PFS, and OS was analysed using the Kaplan–Meier method and Cox regression. All statistical analyses were performed using SPSS (IBM SPSS 20.0, NY, USA). All *P* values are two-sided, and *p* < 0.05 was considered to indicate a statistically significant difference [16].

## Results

### Patient characteristics

Seventy-four AGC patients with a median PFS of 98 days (95% CI, 94 to 102 days) and a median OS of 239 days (95% CI, 208 to 270 days) were involved in this study. The clinical characteristics were listed in Table 1, the percentage for male was 68.9%, while for age over 65 years old was 44.6%. As shown in Table 2, two patients were observed with complete response (CR) and two with partial response (PR), and stable disease (SD) was achieved in nine patients, which resulted in an ORR of 5.4% (95%CI: 0.1% to 10.7%) and DCR of 17.6% (95%CI: 8.7% to 26.4%).

**Table 1:** Characteristics of patients in irAE and non-irAE groups

	Total No. (%)	non-irAE No. (%)	irAE No. (%)	*p*-value
Total N	74	50	24	
Gender				
female	23 (31.1)	12 (24.0)	11 (45.8)	0.067
male	51 (68.9)	38 (76.0)	13 (54.2)	
Age				
< 65	41 (55.4)	26 (52.0)	15 (62.5)	0.460
≥ 65	33 (44.6)	24 (48.0)	9 (37.5)	
ECOG PS				
0	11 (14.9)	5 (10.0)	6 (25.0)	0.159
≥ 1	63 (85.1)	45 (90.0)	18 (75.0)	
Treatment Line				
1–2	25 (33.8)	12 (24.0)	13 (54.2)	0.017
3–4	49 (66.2)	38 (76.0)	11 (45.8)	
Number of Metastatic Sites				
< 2	66 (89.2)	46 (92.0)	20 (83.3)	0.424
≥ 2	8 (10.8)	4 (8.0)	4 (16.7)	
Histologic Type				
non-adenocarcinoma	5 (6.8)	3 (6.0)	2 (8.3)	0.657
adenocarcinoma	69 (93.2)	47 (94.0)	22 (91.7)	
HER2 Status				
negative	66 (89.2)	44 (88.0)	22 (91.7)	1.000
positive	8 (10.8)	6 (12.0)	2 (8.3)	
Disease Status				
stage III	29 (39.2)	20 (40.0)	9 (37.5)	0.757
Stage IV	33 (44.6)	23 (46.0)	10 (41.7)	
recurrence	12 (16.2)	7 (14.0)	5 (20.8)	
ALP				
low	41 (55.4)	27 (54.0)	14 (58.3)	0.805
high	33 (44.6)	23 (46.0)	10 (41.7)	
NLR				
low (< 4)	49 (66.2)	38 (76.0)	11 (45.8)	0.017
high (≥ 4)	25 (33.8)	12 (24.0)	13 (54.2)	
Clinical Baseline Value (Median (IQR))				
WBC	5.82 (4.17, 7.20)	5.62 (4.17, 6.56)	6.56 (4.23, 8.00)	0.145
Neutrophil	3.45 (3.09, 5.13)	3.45 (3.09, 5.03)	5.03 (2.81, 6.72)	0.282
Lymphocyte	0.88 (0.75, 1.34)	1.01 (0.84, 1.76)	0.85 (0.74, 0.96)	0.049
NLR	3.77 (2.57, 4.98)	3.68 (2.57, 3.93)	4.98 (3.26, 7.56)	0.081
Plt	188.00 (135.25, 257.50)	196.50 (90.00, 262.00)	188.00 (148.00, 234.00)	0.826
Hemoglobin	109.00 (101.00, 124.28)	114.00 (107.50, 131.68)	92.00 (76.00, 103.00)	0.001

*Abbreviations*: *ECOG PS* Eastern Cooperative Oncology Group performance status, *NLR* Neutrophilto-lymphocyte ratio, *ALP* Alkaline phosphatase, *WBC* White blood cell, *Plt* Platelet; non-adenocarcinoma, including undifferentiated carcinoma (*n* = 2), adeno-squamous carcinoma (*n* = 2) and squamous carcinoma (*n* = 1)

**Table 2:** Response to immunotherapy

Response	Total	irAE group	Non-irAE group	*p*-value
PD	61	14	47	-
SD	9	6	3	-
PR	2	2	0	-
CR	2	2	0	-
ORR	5.4% (95%CI: 0.1% to 10.7%)	16.7% (95%CI: 4.7% to 37.4%)	-	-
DCR	17.6% (95%CI: 8.7% to 26.4%)	41.7% (95%CI: 20.4% to 62.9%)	6.0% (95%CI: 0.0% to 12.8%)	0.001

*Abbreviations*: *SD* Stable disease, *PR* Partial response, *PD* Progressive disease, *CR* Complete response, *ORR* Objective response rate, *DCR* Disease control rate

### Comparison between irAE and non-irAE groups

As the patient background shows in Table 1, no significant differences in clinical profiles of the irAE group and non-irAE group, apart from treatment line, NLR, the counts of lymphocyte and hemoglobin.

An ORR of 16.7% (95%CI: 4.7% to 37.4%) and DCR of 41.7% (95%CI: 20.4% to 62.9%) was observed for all 24 irAE patients (2 CR, 2 PR, 6 SD), whereas only 3 SD patients with DCR of 6.0% (95%CI: 0.0% to 12.8%) archeived in non-irAE group. The difference in DCR between the two groups was significant (*p* = 0.001).

**Fig. 1:**
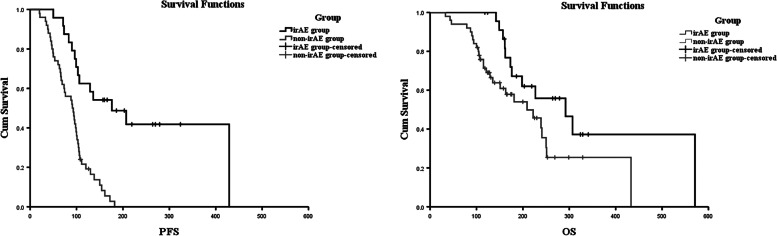
The association of irAEs on the prognosis of AGC patients. **A** The Kaplan–Meier curve of progression free survival (PFS). **B** The Kaplan–Meier curve of overall survival (OS)

The Kaplan–Meier curves of PFS and OS in the irAE and the non-irAE groups are shown in Table 3 and Fig. 1. The median PFS rates with and without irAEs were 176 days (95%CI: 72 to 280 days) and 94 days (95%CI: 77 to 113 days) (Fig. 1A), respectively. Median OS was 292 days (95%CI: 211 to 372 days) in the irAE group and 239 days (95%CI: 152 to 326 days) in the non-irAE group (Fig. 1B). PFS was significantly prolonged in the group that developed irAEs (*p* = 0.001). This survival advantage in irAE group also obtained for OS, but *p* value of OS is at the borderline statistical level (*p* = 0.057).

**Table 3:** Kaplan–Meier survival curve of progression-free survival (PFS) and overall survival (OS)

	Median PFS (days)	*p*-value	Median OS (days)	*p*-value
irAE group	176 (95%CI: 72 to 280)	0.001	292 (95%CI: 211 to 372)	0.057
Non-irAE group	94 (95%CI: 77 to 113)		239 (95%CI: 152 to 326)	

*Abbreviations*: *PFS* Progression-free survival, *OS* Overall survival

In the multivariate analysis for OS using irAEs status, ECOG score, treatment line number, the number of metastatic sites, disease status, ALP and NLR as covariates in these AGC patients, the irAE group were associated with longer OS at statistical levels (HR = 0.269, 95%CI: 0.088 to 0.822, *p* = 0.021) (Table 4).

**Table 4:** Univariate and multivariate analyses of OS with Cox regression models

Covariate	Univariate analysis (*n* = 74)				Multivariate analysis (*n* = 74)			
	Exp(B)	95.0% CI			Exp(B)	95.0% CI		
		Lower	Upper	*P -*value		Lower	Upper	*P -*value
Group								
non-irAE	Reference				Reference			
irAE	0.515	0.254	1.044	0.066	0.269	0.088	0.822	0.021
ECOG PS								
0	Reference				Reference			
≥ 1	1.207	0.502	2.900	0.674	1.418	0.557	3.611	0.464
Treatment line								
1–2	Reference				Reference			
3–4	1.915	0.867	4.228	0.108	1.851	0.697	4.917	0.217
Number of Metastatic Sites								
< 2	Reference				Reference			
≥ 2	1.476	0.557	3.909	0.434	1.806	0.467	6.987	0.392
Disease Status								
stage III-IV	Reference				Reference			
recurrence	1.553	0.726	3.324	0.257	3.252	1.138	9.292	0.028
ALP								
low	Reference				Reference			
high	1.652	0.859	3.177	0.133	2.035	1.022	4.051	0.043
NLR								
low (< 4)	Reference				Reference			
high (≥ 4)	1.506	0.744	3.049	0.255	1.030	0.413	2.570	0.950

*Abbreviations*: *ECOG PS* Eastern Cooperative Oncology Group performance status, *NLR* Neutrophilto-lymphocyte ratio, *ALP* Alkaline phosphatase

In addition, univariate and multivariate analyses were performed on the relationship between different types of irAEs and PFS or OS. We observed that patients with thyroid dysfunction (*p* = 0.013) and rash/pruritus/myalgia/fatigue (*p* = 0.009) had a significantly lower risk of death, associated with longer PFS. The analysis of OS also showed the same trend, but there was no statistical difference (Table 5).

**Table 5:** Relationship between immune-related adverse events and PFS and OS

	**PFS**				**OS**			
	Univariable HR (95% CI)	*P -*value	Multivariable HR (95% CI)	*P -*value	Univariable HR (95% CI)	*P -*value	Multivariable HR (95% CI)	*P -*value
Hypothyroidism/Hyperthyroidism	0.332 (0.131–0.842)	0.020	0.291 (0.110–0.768)	0.013	0.515 (0.199–1.334)	0.172	0.518 (0.197–1.361)	0.182
Diarrhea/Colitis	0.743 (0.181–3.060)	0.681	0.365 (0.083–1.610)	0.183	0.350 (0.048–2.562)	0.301	0.167 (0.020–1.403)	0.099
Rash/Pruritus/Myalgia/Fatigue	0.078 (0.011–0.579)	0.013	0.065 (0.008–0.501)	0.009	0.213 (0.029–1.558)	0.128	0.216 (0.029–1.590)	0.132
Hypohemoglobin/Thrombocytopenia	1.469 (0.356–6.057)	0.595	1.209 (0.290–5.038)	0.794	2.240 (0.573–10.218)	0.229	4.460 (0.935–21.279)	0.061
Damage of liver or kidney function/Myocardial enzyme increased	1.970 (0.710–5.464)	0.193	2.778 (0.994–7.764)	0.051	1.261 (0.376–4.232)	0.707	1.066 (0.314–3.625)	0.918

### Toxicity

The irAEs occurred in twenty-four of 74 patients (32.4%) in our study. Details of these irAEs are shown in Table 6. The most frequent adverse event was hypothyroidism (*n* = 6) and the second is hyperthyroidism (*n* = 5). There was a patient experienced grade 3 diarrhea/colitis related to toripalimab, we stopped the ICIs treatment timely and treat with corticosteroid at the same time (Table S2). We also stopped the ICIs treatment in patients two subtype of irAES as grade 2 hepatic dysfunction and myocardial toxicity.

**Table 6:** Categorization of irAEs

irAEs	No. (%)	Median days to onset	Grade of irAEs, n, 1/2/3/4 (%)
Total	29 (100)	70	20 (68.97)/8 (27.59)/1 (3.44)/0
Hypothyroidism	6 (20.69)	94	5 (83.33)/1 (16.67)/0/0
Hyperthyroidism	5 (17.24)	92	2 (0.40)/3 (0.60)/0/0
Diarrhea/colitis	3 (10.34)	48	2 (66.67)/0/1 (33.33)/0
Rash	3 (10.34)	70	3 (100)/0/0/0
Myocardial enzyme increased	3 (10.34)	21	1 (33.33)/2 (66.67)/0/0
AST/ALT/Bilirubin increased	2 (6.90)	38.5	1 (50)/1 (50)/0/0
Creatinine increased	2 (6.90)	148.5	1 (50)/1 (50)/0/0
Myalgia	1 (3.45)	71	1 (100)/0/0/0
Pruritus	1 (3.45)	26	1 (100)/0/0/0
Thrombocytopenia	1 (3.45)	27	1 (100)/0/0/0
Hypohemoglobin	1 (3.45)	28	1 (100)/0/0/0
Fatigue	1 (3.45)	106	1 (100)/0/0/0

## Discussion

This study showed that irAEs were associated with efficacy of anti-PD-1 antibody in patients with AGC, as determined by favorable prognosis. J. Rogado et al. reported the relation of irAEs with patient characteristics and outcomes in a series of tumors (lung cancer, melanoma, head and neck carcinoma, renal carcinoma, Hodgkin’s lymphoma, urothelial carcinoma and gallbladder adenocarcinoma, hepatocellular carcinoma and Merkel cell carcinoma), the ORR was 16.6% in patients with non-irAEs and 82.5% in patients with irAEs [17]. Our previous study also found that irAEs can also serve as a marker of good prognosis in hepatocellular carcinoma (HCC) [16]. The results that the irAEs associated with better treatment efficiency including DCR, PFS and OS in AGC was consistent with previous reports. But the ORR and DCR in our study were lower than the previous study, it maybe because a large proportion of patients in our study treated with anti-PD-1 antibody in 3–4 treatment line. This means that these patients may have fewer treatment options and poorer physical condition.

We could analyze only a limited number of patient samples, but it is speculated that occurrence of irAEs may be associated with longer PFS and OS. In addition, we found that patients with thyroid dysfunction and skin toxicity had a better prognosis, which is also consistent with previous meta-analysis for non-small cell lung cancer (NSCLC), melanoma and renal cell carcinoma (RCC) treated with anti-PD-1 antibody or anti-CTLA-4 antibody [18]. We were unable to further analyse the relationship between high-grade or low-grade irAEs and prognosis due to the small sample size. The distribution frequency for irAEs and non-irAEs was different referring to treatment lines, but the fact that treatment lines didn’t influence the PFS and OS in our data could basically eliminate the impact on survival due to the distribution difference of irAEs.

Generally, a known prognostic index for AGC based on the clinical trial, Japan Clinical Oncology Group (JCOG) 9912, consists of the following four independent risk factors for survival: performance status ≥ 1, number of metastatic sites ≥ 2, no prior gastrectomy, and elevated alkaline phosphatase (ALP) [19]. Previous studies have reported that NLR in clinical course correlated with prognosis in several cancers [20]. To analyze the impact of known prognostic factors, we adopted these five documented risk factors as covariates for multivariate analysis, we were unable to find an association between these factors and prognosis in the case of statistical differences apart from ALP and the history of gastrectomy. These factors might not display their predictive value in very late advanced GC patient with small sample size.

In our study, we observed predominance in the number of patients with thyroid dysfunction, diarrhea or colitis, and skin toxicity. The distribution characteristics of irAEs were similar to those of previous studies [21]. All irAEs in our study were manageable, and only 1 patient with grade 3 diarrhea/colitis related to toripalimab was observed, which probly due to our baseline examination including the function of relevant organs and related examination every three weeks so as to treat irAEs at early stage. Our results implied irAE particularly low grade irAE was in connection with better efficiency, so we might recommend continuing use for anti-PD-1 antibody therapy in low grade irAE patients.

There is a strong, dynamic and bidirectional link between autoimmunity and cancer. PD-1 interacts with its ligands PD-L1 and PD-L2 to generate negative costimulatory signal through the tyrosine phosphatase SHP2 so as to to weakened T cell activation, which allowing tumor cells to produce immune escape. ICIs can block the binding of PD-1 and PD-L1, increase T cell clones in the tumor microenvironment, and elicit their antitumor effects. The precise mechanisms of irAEs have not been fully uncovered, but they are thought to represent bystander effects from activated T-cells and are consistent with the mechanism of action of ICIs. Specifically, tumors inflamed with cytotoxic T lymphocytes prior to treatment then experience further inflammation and tumor-cell death when treated with ICIs. Similarly, the organs with surface antigens similar to tumors may experience pronounced, clinically apparent inflammation when these key negative regulators of T-cell function are removed [22–24]. However, the mechanisms why some toxicities occur in specific patients, and the relationship between toxicity and response, are not yet apparent.

Although no grade 4–5 irAEs were observed in our study, it is necessary to explore biomarkers of irAEs in order to prevent the occurrence of severe irAEs. Previous studies have suggested that CD8^+^ T cells [25], eosinophils [26], regulatory T cells [27], auto-antibodies [28], inflammatory cytokines (such as interleukin-17) [29], and gut microbiome may be involved in the development of irAEs [30]. However, all biomarkers are controversial due to the limited research data and have not been verified in large-scale prospective clinical studies. The discovery of irAEs is usually detected only by detection of relevant indicators and clinical observation at present.

This study has some limitations. First, the sample size was small and the date were only collected from a single center, it was valuable to collect more data from a multicenter for stratified analysis to identify different types or grades of irAEs responsible for ICI efficiency. Second, we confirmed and evaluated irAEs carefully based on the combined medicine displayed a spectrum of different adverse events compared with ICIs, but the effect of combined medicine on adverse reactions and prognosis in patients was not excluded completely in this study. Third, our study only included AGC patients with no preexisting autoimmune disease, the correlation between irAEs and prognosis in patients with preexisting autoimmune disease needs further exploration. But as far as we know, this is the first work to reveal an association between irAEs and the efficacy in AGC patients who treated with various ICIs and almost all of our patients received combination therapy. The result of our study has confirmed the value for irAEs as a biomarker on treatment efficiency of ICIs and built the foundation for future research with a larger population. It may inspire other researchers to look for predictive values of irAEs in other tumors.

## Conclusions

The occurrence of irAEs was significantly associated with better clinical prognosis of the patients of advanced gastric cancer treated with anti-PD-1 antibody. The mechanism and biomarkers for irAEs should be clarified in the future.

## Supplementary Information

**Additional file 1: Table S1.** Treatment information of overall patients.

**Additional file 2: Table S2.** Clinical information of 24 patients in irAE group.

## Data Availability

The datasets generated during the current study are not publicly available due to ethical restrictions, but are available from the corresponding author on reasonable request.

## Abbreviations

ICIs: Immune checkpoint inhibitors
irAE: Immune-related adverse event
SD: Stable disease
PR: Partial response
PD: Progressive disease
CR: Complete response
ORR: Objective response rate
DCR: Disease control rate
PFS: Progression-free survival
OS: Overall survival
AGC: Advanced gastric cancer
HER-2: Human epidermal growth factor receptor 2
PD-1: Programmed cell death 1
CTLA-4: Cytotoxic T-lymphocyte antigen 4
NSCLC: Non-small-cell lung cancer
RCC: Renal cell carcinoma
ECOG PS: Eastern Cooperative Oncology Group performance status
NLR: Neutrophilto-lymphocyte ratio
CT: Computed tomography
IQR: Interquartile range
ALP: Alkaline phosphatase

## References

[1] Sung H, Ferlay J, Siegel RL, Laversanne M, Soerjomataram I, Jemal A, Bray F (2021). Global Cancer Statistics 2020: GLOBOCAN Estimates of Incidence and Mortality Worldwide for 36 Cancers in 185 Countries. CA Cancer J Clin.

[2] Smyth EC, Verheij M, Allum W, Cunningham D, Cervantes A, Arnold D (2016). Gastric cancer: ESMO Clinical Practice Guidelines for diagnosis, treatment and follow-up. Ann Oncol.

[3] Koizumi W, Narahara H, Hara T, Takagane A, Akiya T, Takagi M, Miyashita K, Nishizaki T, Kobayashi O, Takiyama W (2008). S-1 plus cisplatin versus S-1 alone for first-line treatment of advanced gastric cancer (SPIRITS trial): a phase III trial. Lancet Oncol.

[4] Yamada Y, Higuchi K, Nishikawa K, Gotoh M, Fuse N, Sugimoto N, Nishina T, Amagai K, Chin K, Niwa Y (2015). Phase III study comparing oxaliplatin plus S-1 with cisplatin plus S-1 in chemotherapy-naïve patients with advanced gastric cancer. Annal Oncol.

[5] Postow MA, Sidlow R, Hellmann MD (2018). Immune-Related Adverse Events Associated with Immune Checkpoint Blockade. N Engl J Med.

[6] Kang YK, Boku N, Satoh T, Ryu MH, Chao Y, Kato K, Chung HC, Chen JS, Muro K, Kang WK (2017). Nivolumab in patients with advanced gastric or gastro-oesophageal junction cancer refractory to, or intolerant of, at least two previous chemotherapy regimens (ONO-4538-12, ATTRACTION-2): a randomised, double-blind, placebo-controlled, phase 3 trial. Lancet (London, England).

[7] Boku N, Ryu MH, Kato K, Chung HC, Minashi K, Lee KW, Cho H, Kang WK, Komatsu Y, Tsuda M (2019). Safety and efficacy of nivolumab in combination with S-1/capecitabine plus oxaliplatin in patients with previously untreated, unresectable, advanced, or recurrent gastric/gastroesophageal junction cancer: interim results of a randomized, phase II trial (ATTRACTION-4). Ann Oncol.

[8] Janjigian YY, Shitara K, Moehler M, Garrido M, Salman P, Shen L, Wyrwicz L, Yamaguchi K, Skoczylas T, Campos Bragagnoli A (2021). First-line nivolumab plus chemotherapy versus chemotherapy alone for advanced gastric, gastro-oesophageal junction, and oesophageal adenocarcinoma (CheckMate 649): a randomised, open-label, phase 3 trial. Lancet (London, England).

[9] Shitara K, Van Cutsem E, Bang YJ, Fuchs C, Wyrwicz L, Lee KW, Kudaba I, Garrido M, Chung HC, Lee J (2020). Efficacy and Safety of Pembrolizumab or Pembrolizumab Plus Chemotherapy vs Chemotherapy Alone for Patients With First-line, Advanced Gastric Cancer: The KEYNOTE-062 Phase 3 Randomized Clinical Trial. JAMA Oncol.

[10] Kawazoe A, Fukuoka S, Nakamura Y, Kuboki Y, Wakabayashi M, Nomura S, Mikamoto Y, Shima H, Fujishiro N, Higuchi T (2020). Lenvatinib plus pembrolizumab in patients with advanced gastric cancer in the first-line or second-line setting (EPOC1706): an open-label, single-arm, phase 2 trial. Lancet Oncol.

[11] Peng Z, Wei J, Wang F, Ying J, Deng Y, Gu K, Cheng Y, Yuan X, Xiao J, Tai Y (2021). Camrelizumab Combined with Chemotherapy Followed by Camrelizumab plus Apatinib as First-line Therapy for Advanced Gastric or Gastroesophageal Junction Adenocarcinoma. Clin Cancer Res.

[12] Voskens CJ, Goldinger SM, Loquai C, Robert C, Kaehler KC, Berking C, Bergmann T, Bockmeyer CL, Eigentler T, Fluck M (2013). The price of tumor control: an analysis of rare side effects of anti-CTLA-4 therapy in metastatic melanoma from the ipilimumab network. PLoS One.

[13] Brahmer JR, Lacchetti C, Schneider BJ, Atkins MB, Brassil KJ, Caterino JM, Chau I, Ernstoff MS, Gardner JM, Ginex P (2018). Management of Immune-Related Adverse Events in Patients Treated With Immune Checkpoint Inhibitor Therapy: American Society of Clinical Oncology Clinical Practice Guideline. J Clin Oncol.

[14] Matsuoka H, Hayashi T, Takigami K, Imaizumi K, Shiroki R, Ohmiya N, Sugiura K, Kawada K, Sawaki A, Maeda K (2020). Correlation between immune-related adverse events and prognosis in patients with various cancers treated with anti PD-1 antibody. BMC Cancer.

[15] Masuda K, Shoji H, Nagashima K, Yamamoto S, Ishikawa M, Imazeki H, Aoki M, Miyamoto T, Hirano H, Honma Y (2019). Correlation between immune-related adverse events and prognosis in patients with gastric cancer treated with nivolumab. BMC Cancer.

[16] Xu S, Lai R, Zhao Q, Zhao P, Zhao R, Guo Z (2021). Correlation between immune-related adverse events and prognosis in hepatocellular carcinoma patients treated with immune checkpoint inhibitors. Front Immunol.

[17] Rogado J, Sánchez-Torres JM, Romero-Laorden N, Ballesteros AI, Pacheco-Barcia V, Ramos-Leví A, Arranz R, Lorenzo A, Gullón P, Donnay O (2019). Immune-related adverse events predict the therapeutic efficacy of anti-PD-1 antibodies in cancer patients. Eur J Cancer.

[18] Zhou X, Yao Z, Yang H, Liang N, Zhang X, Zhang F (2020). Are immune-related adverse events associated with the efficacy of immune checkpoint inhibitors in patients with cancer? A systematic review and meta-analysis. BMC Med.

[19] Takahari D, Boku N, Mizusawa J, Takashima A, Yamada Y, Yoshino T, Yamazaki K, Koizumi W, Fukase K, Yamaguchi K (2014). Determination of prognostic factors in Japanese patients with advanced gastric cancer using the data from a randomized controlled trial, Japan clinical oncology group 9912. Oncologist.

[20] Nakamura Y, Tanaka R, Maruyama H, Ishitsuka Y, Okiyama N, Watanabe R, Fujimoto M, Fujisawa Y (2019). Correlation between blood cell count and outcome of melanoma patients treated with anti-PD-1 antibodies. Jpn J Clin Oncol.

[21] Wang PF, Chen Y, Song SY, Wang TJ, Ji WJ, Li SW, Liu N, Yan CX (2017). Immune-Related Adverse Events Associated with Anti-PD-1/PD-L1 Treatment for Malignancies: A Meta-Analysis. Front Pharmacol.

[22] Das S, Johnson DB (2019). Immune-related adverse events and anti-tumor efficacy of immune checkpoint inhibitors. J Immunother Cancer.

[23] Passat T, Touchefeu Y, Gervois N, Jarry A, Bossard C, Bennouna J (2018). Physiopathological mechanisms of immune-related adverse events induced by anti-CTLA-4, anti-PD-1 and anti-PD-L1 antibodies in cancer treatment. Bull Cancer.

[24] Yoest JM (2017). Clinical features, predictive correlates, and pathophysiology of immune-related adverse events in immune checkpoint inhibitor treatments in cancer: a short review. ImmunoTargets and therapy.

[25] Valpione S, Pasquali S, Campana LG, Piccin L, Mocellin S, Pigozzo J, Chiarion-Sileni V (2018). Sex and interleukin-6 are prognostic factors for autoimmune toxicity following treatment with anti-CTLA4 blockade. J Transl Med.

[26] Jaber SH, Cowen EW, Haworth LR, Booher SL, Berman DM, Rosenberg SA, Hwang ST (2006). Skin reactions in a subset of patients with stage IV melanoma treated with anti-cytotoxic T-lymphocyte antigen 4 monoclonal antibody as a single agent. Arch Dermatol.

[27] Selby MJ, Engelhardt JJ, Quigley M, Henning KA, Chen T, Srinivasan M, Korman AJ (2013). Anti-CTLA-4 antibodies of IgG2a isotype enhance antitumor activity through reduction of intratumoral regulatory T cells. Cancer Immunol Res.

[28] Toi Y, Sugawara S, Sugisaka J, Ono H, Kawashima Y, Aiba T, Kawana S, Saito R, Aso M, Tsurumi K (2019). Profiling Preexisting Antibodies in Patients Treated With Anti-PD-1 Therapy for Advanced Non-Small Cell Lung Cancer. JAMA Oncol.

[29] Tarhini AA, Zahoor H, Lin Y, Malhotra U, Sander C, Butterfield LH, Kirkwood JM (2015). Baseline circulating IL-17 predicts toxicity while TGF-β1 and IL-10 are prognostic of relapse in ipilimumab neoadjuvant therapy of melanoma. J Immunother Cancer.

[30] Vétizou M, Pitt JM, Daillère R, Lepage P, Waldschmitt N, Flament C, Rusakiewicz S, Routy B, Roberti MP, Duong CP (2015). Anticancer immunotherapy by CTLA-4 blockade relies on the gut microbiota. Science (New York, NY).

