# Supplementary material for: Are anti-PD-1-associated immune related adverse events a harbinger of favorable clinical prognosis in patients with gastric cancer?
Source: BMC Cancer. 2022 Nov 5;22:1136. doi: 10.1186/s12885-022-10199-x (PMC9636611; doi:10.1186/s12885-022-10199-x)
Supplement: Supplementary file 1 — Additional file 1: Table S1. Treatment information of overall patients. [file 12885_2022_10199_MOESM1_ESM.pdf]

Table S1 Treatment information of overall patients

Treatment Line	Therapeutic Schedule			The number of people
	ICIs	Targeted Medicine	Chemotherapeutic Regimen	
1	Tislelizumab	Apatinib	-	2
1	Tislelizumab	Lenvatinib	Paclitaxel (Albumin Bound) and Lobaplatin	1
2	Pembrolizumab	Lenvatinib	-	2
2	Camrelizumab	Apatinib	-	10
2	Camrelizumab	Apatinib	Paclitaxel (Albumin Bound)	2
2	Camrelizumab	Trastuzumab	Paclitaxel (Albumin Bound)	2
2	Toripalimab	Apatinib	-	2
2	Sintilimab	Apatinib	-	1
2	Sintilimab	Apatinib	Lobaplatin	1
2	Sintilimab	Trastuzumab	Tegafur Gimeracil Oteracil Potassium Capsule	2
3	Camrelizumab	Apatinib	-	19
3	Camrelizumab	Lenvatinib	-	1
3	Pembrolizumab	Apatinib	-	1
3	Pembrolizumab	Trastuzumab	Paclitaxel (Albumin Bound)	2
3	Toripalimab	Apatinib	-	2
3	Tislelizumab	-	-	2
3	Tislelizumab	-	Capecitabine	1
3	Sintilimab	Apatinib	-	15
3	Sintilimab	Trastuzumab	Tegafur Gimeracil Oteracil Potassium Capsule	2
4	Camrelizumab	Apatinib	-	1
4	Pembrolizumab	Apatinib	-	1
4	Toripalimab	Apatinib	-	2
