# Supplementary material for: Are anti-PD-1-associated immune related adverse events a harbinger of favorable clinical prognosis in patients with gastric cancer?
Source: BMC Cancer. 2022 Nov 5;22:1136. doi: 10.1186/s12885-022-10199-x (PMC9636611; doi:10.1186/s12885-022-10199-x)
Supplement: Supplementary file 2 — Additional file 2: Table S2. Clinical information of 24 patients in irAE group. [file 12885_2022_10199_MOESM2_ESM.pdf]

Table S2 Clinical information of 24 patients in irAE group

	irAEs	Grades	Time (days)	Treatment line	Therapeutic Schedule			Treatment	Discontinued
					ICIs	Targeted Medicine	Chemotherapeutic Regimen		
1	AST/ALT/Bilirubin increased	1	53	4	Pembrolizumab	Apatinib	-	protect liver treatment	no
2	Hypothyroidism	1	106	2	Sintilimab	Apatinib	-	observed	no
3	Diarrhea/colitis	1	28	4	Toripalimab	Apatinib	-	observed	no
4	Hypothyroidism	1	63	1	Tislelizumab	Apatinib	-	observed	no
5	AST/ALT/Bilirubin increased	2	24	2	Camrelizumab	Apatinib	-	protect liver treatment	yes
6	Hyperthyroidism	1	92	2	Camrelizumab	Trastuzumab	Paclitaxel (Albumin Bound)	observed	no
7	Thrombocytopenia	1	27	3	Toripalimab	Apatinib	-	observed	no
	Diarrhea/colitis	1	48						
8	Creatinine increased	2	147	3	Pembrolizumab	Apatinib	-	improved renal function	no
9	Myocardial enzyme increased	2	21	3	Sintilimab	Apatinib	-	observed	yes
10	Creatinine increased	1	150	2	Toripalimab	Apatinib	-	improved renal function	no
11	Hypothyroidism	1	126	2	Camrelizumab	Apatinib	-	observed	no
12	Hyperthyroidism	2	146	3	Camrelizumab	Apatinib	-	observed	no
13	Rash	1	71	3	Sintilimab	Trastuzumab	Tegafur Gimeracil Oteracil Potassium Capsule	observed	no
14	Rash	1	70	2	Camrelizumab	Apatinib	-	observed	no
	Hypothyroidism	1	82						
15	Fatigue	1	106	2	Sintilimab	Apatinib	Lobaplatin	observed	no
16	Hypohemoglobin	1	28	4	Toripalimab	Apatinib	-	observed	no
17	Hypothyroidism	1	63	1	Tislelizumab	Apatinib	-	observed	no
18	Hyperthyroidism	1	92	2	Camrelizumab	Apatinib	-	observed	no
	Pruritus	1	26						
19	Diarrhea/colitis	3	48	3	Toripalimab	Apatinib	-	corticosteroid	yes
20	Myocardial enzyme increased	2	147	2	Pembrolizumab	Lenvatinib	-	observed	yes
21	Myocardial enzyme increased	1	21	3	Sintilimab	Apatinib	-	observed	no
22	Hyperthyroidism	2	66	2	Toripalimab	Apatinib	-	observed	no
	Hypothyroidism	1	150						
23	Hyperthyroidism	2	146	3	Camrelizumab	Apatinib	-	observed	no
24	Myalgia	1	71	3	Sintilimab	Apatinib	-	observed	no
	Rash	1	20				-		
